# Physical activity and sedentary behaviour typologies of 10-11 year olds

**DOI:** 10.1186/1479-5868-7-59

**Published:** 2010-07-28

**Authors:** Russell Jago, Kenneth R Fox, Angie S Page, Rowan Brockman, Janice L Thompson

**Affiliations:** 1 Department of Exercise, Nutrition & Health Sciences, University of Bristol, Bristol, UK

## Abstract

**Background:**

Targeted interventions may be more effective at increasing children's physical activity. The aim of this study was to identify clusters of children based on physical activity and sedentary patterns across the week.

**Methods:**

Participants were 761, 10-11 year old children. Participant's self-reported time spent in eight physical activity and sedentary contexts and wore an accelerometer. Cluster analysis was conducted on the time spent in the self-reported physical activity and sedentary contexts. Mean minutes of accelerometer derived of moderate to vigorous physical activity (MVPA) and sedentary time were derived for the entire week, weekdays only, weekend days and four different time periods across each type (weekend or weekday) of days. Differences in the physical activity patterns of the groups derived from the cluster analysis were assessed for overall physical activity as well as for the four time periods on weekdays and weekend days.

**Results:**

Three clusters emerged: 1) High active/Low sedentary; 2) Low active/Moderate sedentary; and 3) High Active/High sedentary. Patterns of activity differed across the week for each group and the High Active/High sedentary obtained the most minutes of MVPA.

**Conclusions:**

Patterns of physical activity and sedentary time differed across the week for each cluster. Interventions could be targeted to the key periods when each group is inactive.

## Background

Regular physical activity is associated with lower levels of several cardiometabolic [1] risk factors among youth [2]. Physical activity is also associated with lower body mass and higher levels of psychological well-being [3-5]. Many youth do not meet the current public health recommendation of an hour of moderate to vigorous physical activity on most days of the week [5-9]. As such there is a need to develop methods of increasing youth physical activity. Current efforts in this regard have had limited success, with effective changes achieved in smaller sub-groups or only in the short-term [10,11].

One reason for the relative lack of success at changing children's physical activity could be that many interventions aim to maximise reach by delivering the same intervention to all children in a school or year group [12]. While this approach increases the number of children exposed to the intervention and therefore has the potential to maximise its public health impact, it fails to target the children with the lowest levels of physical activity or those that spend a lot of time being sedentary.

Children's physical activity is inherently complex, involving a mixture of school-based activities, organised team sports and unstructured play [9]. Similarly, sedentary time is not just periods of inactivity but a product of time spent in specific sedentary behaviours such as TV viewing, playing a games console or talking on the phone [13,14]. In order to understand the best methods of promoting physical activity and screen-viewing we need to understand how these behaviours are related. For example, some very active boys take part in sustained periods of physical activity during team sports and then spend the rest of the day watching TV and playing video games [9]. Other children may spend a similar amount of time watching TV and playing video games but are largely inactive at all other times. Both of these groups spend a lot of time engaged in sedentary behaviours but the approach to promoting physical activity in the two groups is very different. Understanding how sedentary and physical activity behaviours are clustered among children may provide us with useful information about how to tailor interventions to specific groups.

The promotion of children's physical activity has been hampered by the implicit assumption that physical activity is uniform with "active" children engaging in more physical activity than their "sedentary" counterparts throughout the day and across the week. Furthermore, a singular approach does not consider the time of day when children are sedentary or active. Children may accumulate activity in different contexts at different times of day [9,15]. For example, if we assume that "sedentary" children are less active across the day we might assume that they are less active than their "active" peers because they do less physical activity during school break periods and physical education lessons. However, it is highly plausible that the activity levels of both groups are comparable during break periods and physical education lessons, but the extra activity of the "active" group is a function of physical activity after school and at the weekends. In this scenario interventions to increase physical activity during break periods and physical education classes might yield small increases for both groups but will not deliver the necessary increases in the "sedentary" children's physical activity patterns after school. Thus, in order to design effective interventions we need to understand the temporal patterning of children's physical activity patterns across the week.

In light of the issues outlined above this paper will address three inter-linked research questions. 1) Are there clusters of 10-11 year old children who share similar physical activity and sedentary behaviours? 2) If so, what are the characteristics of the cluster groups and how could they be identified for future interventions? 3) Are there differences in the levels and temporal patterns of physical activity among the cluster groups?

## Methods

### Sampling and participants

Data are from the Bristol Parent, Peers and Physical Activity (Bristol 3Ps) study http://www.bris.ac.uk/enhs/research/projects/bristol3ps.html. Details of the overall study design have been reported elsewhere [16]. Briefly, participants were 10-11 year old children recruited from 40 primary schools in Bristol, UK. Sampling was performed based on the Index of Multiple Deprivation (IMD) score for primary school location. The IMD is an area level measure of deprivation that includes income, health, educational and employment status [17] with higher scores indicating higher levels of deprivation i.e. lower socioeconomic status (SES). Schools were randomly recruited from tertiles of school IMD within a 15 mile radius of the University of Bristol. In total, 1684 Year 6 children were invited to take part of which 1026 provided consent (60.9%). The recruitment rate was 61% in High SES schools, 65% in Middle SES schools and 60% in Low SES schools. Of the 1026 students who provided consent, 986 (58.5% of possible sample) provided some data, with the remaining students absent on data collection days. 952 children provided complete data for the eight questions that were used to conduct the cluster analysis and of these 761 students provided at least 3 days of valid accelerometer data and were used in the combined cluster and accelerometer analyses. Data were collected between April 2008 and March 2009. This study was approved by a University of Bristol ethics committee and informed parental consent was obtained for all participants.

### Procedures

Participants self-reported responses to eight questions that assessed time spent in specific sedentary and physical activity contexts. Participants were asked to report the usual hours per day spent: 1) Watching TV; 2) Using a computer (except for homework); 3) Using a phone or texting and 4) Using a games console or other video game device. Response categories for each question were none, 1-2 (>1 but <2), 2-3 (>2 but <3), 3-4 (>3 but <4), 4-5 (>4 but <5) and 5 or more hours per day. We used this question format as parental responses for this question have been shown to correlate (r = 0.60) with the data from 10 days of TV diaries when assessing the behavior of 5 year old children [18], which is the highest validity of current methodologies [19]. This approach has also been used very successfully as a self-report item among European children and adolescents [20]. Participants were also asked to report how often they: 1) attended sport or exercise clubs at school; 2) attended sport or exercise clubs outside of school; 3) played with friends or family members outside near the home and 4) played with friends or family members in the home or garden per week. Response options for these questions were: never, 1-2 days per week, 3-4 days per week and 5 or more days per week.

Physical activity volume and intensity and sedentary time were assessed using GT1 M accelerometers (ActiGraph, LLC, Pensacola, FL) which were set to record every 10 seconds. All participants were provided with instructions on wearing the monitor and data were collected for five complete days. Height was measured using a SECA Leicester stadiometer (HAB International, Northampton) and weight using a SECA digital scale (HAB International, Northampton). Body mass index (kg/m^2^) was calculated and converted to an age and gender specific standard deviation score (BMI SDS) [21]. Participant address including postcode was obtained via parental report and used to derive the IMD score for the child's primary residence.

#### Accelerometer data processing

Periods in which ≥60 minutes of zero counts were obtained were interpreted as time when the monitor was not worn; these periods were removed from the analysis [22]. Each day of accelerometer data was considered valid if data were obtained for at least 500 minutes [23]. Participants were included in aggregate analysis if they provided ≥3 days of valid accelerometer data. To provide a measure of the overall volume of physical activity in which the participants engaged, mean accelerometer counts per minute (Mean CPM) were calculated. To provide an indication of the intensity of the participants' physical activity the mean minutes spent sedentary (Sed Minutes) and engaged in moderate to vigorous intensity physical activity (MVPA minutes) per day were obtained using established thresholds of ≤799 cpm for sedentary time and ≥3200 cpm for MVPA [24]. The thresholds we used were however determined using the older 7164 version of the Actigraph accelerometer. Thus, as the GT1 M monitors provide values that are 9% lower [25], the threshold was corrected by a factor of 0.91 to 2912 cpm for MVPA and 727com for sedentary minutes. We acknowledge that there is no consensus on the most appropriate accelerometer thresholds for children [26] but we opted for the Puyau thresholds because they were derived from a sample of similar aged children using whole body calorimetry [24].

To provide a temporal context for the accelerometer data, four time periods were used to categorise data across the day. To facilitate comparisons and ensure consistency the same periods were used for weekdays and weekend days. "Early morning" was from 6:00 am until 8:59 am and was designed to capture physical activity before school. "Core hours" were from 9:00 am until 2:59 pm and this was a period designed to correspond to school hours. "Afternoon" was from 3:00 pm until 5:59 pm and was designed to capture activity after school. The final period was the evening from 6:00 pm until 8:59 pm, which was designed to capture evening physical activity. Mean CPM as well as mean MVPA and sedentary minutes were obtained for each period for both weekdays and weekend days. Participants were included in the weekday analyses if they had a minimum of 2 days of valid data and were included in the weekend days if they had a minimum of 1 day of valid weekend accelerometer data.

#### Analysis

Descriptive statistics (means, standard deviations and percents) were calculated for all variables. To generate groups of children who had similar characteristics, partition based cluster analysis was applied using SPSS (version 16) to responses on the eight sedentary and physical activity behavioural questions. A goal of this approach is to group participants with similar behavioural profiles together whilst also maximizing the variability between the clusters [27]. Three non-overlapping clusters were obtained and used in all subsequent analyses. Analysis of variance and χ^2 ^tests were used to identify if the BMI percentile, IMD or gender of the participants differed across the three clusters.

In order to examine if overall physical activity patterns differed by cluster group, analysis of variance tests were used with Mean CPM, Mean MVPA or Mean Sedentary minutes per day as the outcome and cluster group as a factor. Significant main effects were further explored using Scheffé pair-wise comparison tests. To examine if differences were still evident after adjustment for key confounders a linear regression model, in which the cluster groups were the main exposure variables and Mean CPM was the outcome variable, was then run with the models adjusted for gender, BMI percentile and household IMD. This process was then repeated for Mean Sedentary minutes and Mean MVPA minutes.

To further examine if there were differences across the weekday and weekend time periods analysis of variance tests were repeated for each of the four weekday and weekend time periods for all three accelerometer variables. Linear regression models that were comparable to those described above were then used to examine any association that yielded a statistically significant (p < .05) main effect in the analysis of variance tests. All analysis of variance tests and regression models were performed in STATA (version 10.1, College Station, TX) and the R^2 ^for the overall model was obtained. Robust standard errors were used to take account of clustering of participants in schools (i.e. non-independence of pupils from the same school), in the computation of 95% confidence intervals and p-values. Alpha was set at 0.05.

## Results

The results of the cluster analysis are presented in Table 1 for the 952 participants that provided complete responses for the eight screening questions. Three distinct clusters emerged. The first group (n = 359) self-reported high participation in the four activity behaviours and lower amounts of time in sedentary pursuits than the other two groups and has therefore been termed the "High Active/Low Sedentary group (Hi-Act/Low-Sed). The second cluster (n = 436) are characterised by low levels of physical activity and moderate time in sedentary behaviours and have therefore been termed the "Low Active/Medium Sedentary group" (Lo-Act/Med-Sed). The third cluster (n = 157) have high levels of both physical activity and sedentary time and are therefore the "High Activity/High Sedentary (Hi-Act/Hi-Sed) group. Analysis of variance tests on the characteristics of the cluster members indicated that there were no significant differences in either mean BMI SDS (F = 0.76, p = 0.469) or the mean IMD score (F = 1.65, p = 0.192) of the clusters. There was a significant difference in the proportion of males and females in the clusters (χ^2 ^= 1.25, p = 0.003) with a higher proportion of males (40.8% vs. 35.3) in the Hi-Act/Low-Sed and Hi-Act/Hi-Sed (19.3 vs. 13.9%) groups and more girls (50.5%) than boys (39.9%) in the Lo-Act/Med-Sed group.

**Table 1:** Descriptive characteristics of participants in each of the three cluster groups

	Hi-Act/Low-Sed (n = 359)		Lo-Act/Med-Sed (n = 436)		Hi-Act/Hi-Sed (n = 157)	
**Variable**	**Mean**	**SD**	**Mean**	**SD**	**Mean **	**SD**

Hours per day of TV	2.08	1.11	2.64	1.27	4.00	1.50

Hours per day of computer time	1.40	0.92	1.50	1.11	3.70	1.67

Hours per day using a phone or texting	0.84	0.72	0.74	0.79	2.42	1.92

Hours per day using a games console	1.45	0.90	1.45	1.03	3.74	1.67

Participation in sports clubs at school	1.45	0.92	0.68	0.61	1.31	0.98

Participation in sport clubs outside of school	1.55	0.98	0.64	0.64	1.16	0.99

Playing with friends near home	2.07	0.87	1.30	0.78	1.92	0.99

Playing in home or garden	2.06	0.80	0.91	0.59	1.50	1.01

						

Group characteristics	**Mean**	**SD**	**Mean**	**SD**	**Mean **	**SD**

Age (years)						

BMI SDS	0.43	1.09	0.49	1.16	0.57	1.32

IMD Score	20.84	15.76	20.73	16.52	23.46	17.89

						

	**Female n (%)^1^**	**Male n (%)**	**Female n (%)**	**Male n (%)**	**Female n (%)**	**Male n (%)**

Gender	178 (35.3)	178 (40.8)	255 (50.5)	174 (39.9)	71 (13.9)	84 (19.3)

^1 ^% corresponds to percentage of each gender

**Figure 1:**
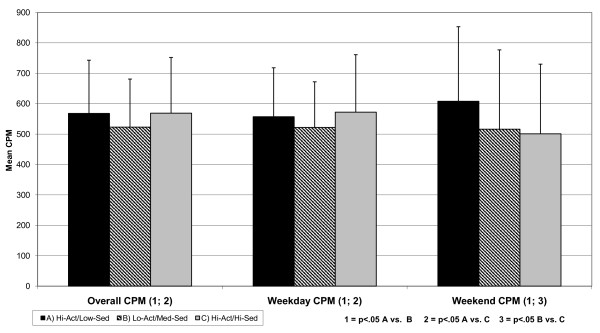
**Mean CPM overall and for weekdays and weekend days by cluster group**.

Figure 1 shows the mean overall accelerometer CPMs together with standard deviations for each group. Mean CPM differed by cluster group with the Hi-Act/Low-Sed group engaging in a higher volume of activity than the Lo-Act/Med-Sed group (568.3 vs. 523.4, p = .004); the Hi-Act/Hi-Sed group also engaged in a higher volume of activity than the Lo-Act/Med-Sed group (569.3 vs. 523.4, p = .034). Similar patterns were evident when limited to just weekdays with the Hi-Act/Low-Sed group engaging in a higher volume of activity than the Lo-Act/Med-Sed group (557.8 vs. 522.1, p = .038); the Hi-Act/Hi-Sed group also engaged in a higher volume of activity than the (572.3 vs. 522.1, p = .030) Lo-Act/Med-Sed group (data not in figure form). For weekend CPM the Hi-Act/Low-Sed group engaged in a greater volume of activity than the Lo-Act/Med-Sed group (608.4 vs. 515.9, p = .001) with the Lo-Act/Med-Sed group also less active than the Hi-Act/Hi-Sed group (515.9 vs. 500.7 cpm, p = .009).

**Figure 2:**
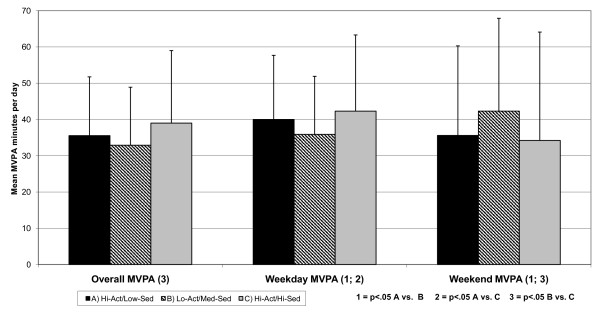
**Minutes MVPA overall and for weekday and weekend days by cluster group**.

Figure 2 shows the mean minutes of MVPA per day together with standard deviations of each group. For overall MVPA, the Hi-Act/Hi-Sed engaged in more MVPA than the Lo-Act/Med-Sed group (39.0 vs. 32.9, p = .002). For weekday MVPA both the Hi-Act/Low-Sed (40.0) and Hi-Act/Hi-Sed (42.3) groups were higher then Lo-Act/Med-Sed group (35.86, p = .024 and .007 respectively). There were no differences in weekend MVPA and no differences in overall sedentary minutes at any assessment.

The regression models predicting overall as well as weekday and weekend specific accelerometer-determined physical activity are presented in Table 2. The Lo-Act/Med-Sed group obtained 40 fewer counts per minute than the Hi-Act/Low-Sed group (t = -3.41, p = 0.002) in a model that accounted for 10.1% of the variance. Similarly, the Lo-Act/Med-Sed group spent an average of 17.35 more minutes per day engaged in sedentary time (t = 2.20, p = .034) than the Hi-Act/Low-Sed group but the model accounted for less than 3% of the overall variance.

**Table 2:** Regression models of differences in physical activity by cluster group for all days and for weekdays and weekend days

			Overall					
	**Coeff**	**t**	**95% CI**	**P**	**Coeff**	**t**	**95% CI**	**P**

**Mean CPM**								

Lo-Act/Med-Sed (ref Hi-Act/Low-Sed)	-40.02	-3.41	-63.77 to -16.26	**0.002**				

Hi-Act/Hi-Sed	-10.10	-0.51	-49.94 to 29.73	0.611				

			**Model R2**	**0.101**				

**Mean Sed Minutes**								

Lo-Act/Med-Sed (ref Hi-Act/Low-Sed)	17.35	2.20	1.43 to 33.28	**0.034**				

Hi-Act/Hi-Sed	13.21	1.31	-7.26 to 33.67	0.199				

			**Model R2**	**0.026**				

**Mean MVPA Minutes**								

Lo-Act/Med-Sed (ref Hi-Act/Low-Sed)	-1.63	-1.44	-3.94 to 0.67	0.159				

Hi-Act/Hi-Sed	3.29	1.50	-1.15 to 7.73	0.143				

			**Model R2**	**0.153**				

	**Weekdays**						**Weekend**	

**Mean CPM**								

Lo-Act/Med-Sed (ref Hi-Act/Low-Sed)	-25.64	-2.18	-49.38 to -1.89	**0.035**	-91.82	-2.97	-154.44 to -29.21	**0.005**

Hi-Act/Hi-Sed	2.44	0.12	-37.24 to 42.11	0.902	-119.40	-3.30	-192.64 to -46.17	**0.002**

			**Model R2**	0.130			**Model R2**	**0.051**

**Mean Sed Minutes**								

Lo-Act/Med-Sed (ref Hi-Act/Low-Sed)	10.06	2.52	1.99 to 18.12	**0.016**	18.30	0.86	-24.70 to 61.30	0.395

Hi-Act/Hi-Sed	14.11	2.01	-0.08 to 28.31	0.051	2.18	0.10	-41.96 to 46.31	0.921

			**Model R2**	**0.051**			**Model R2**	**0.015**

**Mean MVPA Minutes**								

Lo-Act/Med-Sed (ref Hi-Act/Low-Sed)	-3.07	-2.65	-5.42 to -0.73	**0.012**	-1.80	-0.96	-5.59 to 1.99	0.342

Hi-Act/Hi-Sed	1.72	0.82	-2.53 to 5.99	0.417	-1.84	-0.44	-10.23 to 6.54	0.659

			**Model R2**	**0.185**			**Model R2**	**0.059**

All models are adjusted for gender, BMI percentile, and Index of Multiple Deprivation for household

When the models were re-run for weekday accelerometer-determined activity the Lo-Act/Med-Sed group had lower mean CPM (-25.64, p = 0.035) and higher sedentary minutes (10.06, p = 0.016) than the Hi-Act/Low-Sed group. The Lo-Act/Med-Sed group also obtained a little over three fewer minutes of MVPA per day than the Hi-Act/Low-Sed group (t = -2.65, p = 0.012) in a model that accounted for 18% of the variance. When the models were run for weekend physical activity, the Lo-Act/Med-Sed group (-91.82, p = .005) and Hi-Act/Hi-Sed (-119.4, p = 0.002) had lower Mean CPM than the Hi-Act/Low-Sed group.

The analysis of variance tests of differences in activity across the four windows of time are presented for both weekday and weekend physical activity in Table 3. The Hi-Act/Hi-Sed group had higher Mean CPM during core hours on weekdays than the Hi-Act/Low-Sed group (556.6 vs. 493.9, p = .003) and the Lo-Act/Med-Sed group (482.2, p < .001), with the same pattern also evident for minutes of MVPA. On weekdays the Hi-Act/Low-Sed group had fewer sedentary minutes during the afternoon (118.2 vs. 125.7, p = .010) and in the evening (110.9 vs. 108.9 p = .026) than the Lo-Act/Med-Sed group. On the weekend the Hi-Act/Low-Sed group obtained a higher Mean CPM, fewer sedentary minutes and more MVPA minutes than the Lo-Act/Med-Sed group during core hours (all p < .05).

**Table 3:** ANOVA's of differences in activity by cluster by time of day and day of the week

	Hi-Act/Low-Sed		Lo-Act/Med-Sed (n = 436)		Hi-Act/Hi-Sed				
Variable	Mean	SD	Mean	SD	Mean	SD	F	P	Scheffe
Weekday CPM - Early morning	633.93	405.51	624.94	356.48	656.45	320.08	0.33	0.716	

Weekday CPM - Core hours	493.91	163.70	482.24	162.10	556.59	209.97	8.81	**<0.001**	**B, C**

Weekday CPM - Afternoon	723.06	374.11	655.67	323.04	726.05	365.20	3.46	**0.032**	

Weekday CPM - Evening	517.40	334.14	455.83	302.94	532.10	373.31	3.87	**0.021**	

Weekday Sed Mins - Early morning	57.09	28.73	55.82	25.15	53.87	27.51	0.62	0.539	

Weekday Sed Mins - Core hours	275.53	59.17	282.45	46.36	277.85	33.94	1.52	0.220	

Weekday Sed Mins - Afternoon	118.22	35.77	125.73	26.87	121.75	28.22	4.62	**0.010**	**A**

Weekday Sed Mins - Evening	100.91	40.75	108.95	34.04	105.90	34.06	3.69	**0.026**	**A**

Weekday MVPA Mins - Early morning	4.27	3.83	4.25	3.63	4.58	4.11	0.37	0.692	

Weekday MVPA Mins - Core hours	16.74	9.06	16.15	8.66	20.31	11.52	9.20	**<0.001**	**B, C**

Weekday MVPA Mins - Afternoon	11.08	7.90	10.21	6.84	12.29	8.91	3.54	**0.030**	**C**

Weekday MVPA Mins - Evening	5.42	5.27	4.83	4.64	6.09	5.23	3.12	**0.045**	

									

Weekend CPM - Early morning	573.55	710.90	462.18	568.99	401.28	304.41	2.07	0.127	

Weekend CPM - Core hours	641.29	377.41	543.22	340.71	559.44	307.62	5.49	**0.004**	**A**

Weekend CPM - Afternoon	681.19	498.67	608.68	465.76	560.67	369.21	2.81	0.061	

Weekend CPM - Evening	474.10	513.14	392.26	301.46	361.85	253.66	4.12	**0.017**	

Weekend Sed Mins - Early morning	22.78	33.56	20.82	29.79	22.27	36.56	0.26	0.769	

Weekend Sed Mins - Core hours	225.40	68.93	241.38	61.81	228.98	56.44	4.46	**0.012**	**A**

Weekend Sed Mins - Afternoon	122.25	40.51	132.52	30.30	132.63	31.48	6.62	**0.001**	**A, B**

Weekend Sed Mins - Evening	113.67	44.50	119.31	39.96	121.00	44.27	1.59	0.205	

Weekend MVPA Mins - Early morning	1.25	4.64	0.72	1.99	0.76	1.89	1.91	0.149	

Weekend MVPA Mins - Core hours	18.38	16.60	15.01	14.32	15.05	13.92	3.69	**0.026**	**A**

Weekend MVPA Mins - Afternoon	9.76	9.15	8.62	8.21	8.44	8.07	1.45	0.235	

Weekend MVPA Mins - Evening	4.65	5.25	4.32	5.25	4.32	6.08	0.29	0.745	

A = Active vs. Screen-viewing p < .005 B = Active vs. Sedentary p < .005 C = Screen-viewing vs. Sedentary p < .005

The weekday period specific regression models are presented in Table 4. After adjustment for confounders the Lo-Act/Med-Sed group engaged in an average of 53.6 fewer counts per minute than the ref Hi-Act/Low-Sed group during core hours (t = 3.00, p = 0.005) in a model that accounted for 15.9% of the variance. For weekday evening CPM the Lo-Act/Med-Sed group were less active (-63.34, t = -2.05, p = 0.047) but the model accounted for less than 2% of the variance in the outcome. Both the Lo-Act/Med-Sed group (10.9, t = 4.10, p < 0.001) and the Hi-Act/Hi-Sed group (10.4, t = 2.41, p = 0.021) had more sedentary minutes than the ref Hi-Act/Low-Sed group during the afternoon but the model only accounted for 4.6% of the variance. The Lo-Act/Med-Sed group obtained 9.8 more sedentary minutes than the Hi-Act/Low-Sed group on weekday evenings (t = 3.68. p = 0.001). The Hi-Act/Hi-Sed group obtained 3.3 more minutes of MVPA during weekday core hours than the Hi-Act/Low-Sed group (t = 3.42, p = 0.001) in a model that accounted for 16.0% of the variance.

**Table 4:** Regression models of differences in physical activity by cluster group for weekday and weekend windows of time

Weekday	Coeff	t	95% CI	P	Model R2
**CPM - Core hours**					

Lo-Act/Med-Sed (ref Hi-Act/Low-Sed)	-4.58	-0.27	-38.28 to 29.11	0.785	**0.159**

Hi-Act/Hi-Sed	53.64	3.00	17.47 to 89.80	0.005	

**CPM - Afternoon**					

Lo-Act/Med-Sed (ref Hi-Act/Low-Sed)	-51.60	-1.68	-114.72 to 10.73	0.102	**0.033**

Hi-Act/Hi-Sed	-8.57	-0.17	-122.93 to 95.80	0.869	

**CPM - Evening**					

Lo-Act/Med-Sed (ref Hi-Act/Low-Sed)	-63.34	-2.05	-125.88 to -0.79	0.047	**0.016**

Hi-Act/Hi-Sed	-14.86	-0.38	-94.10 to 64.38	0.706	

**Sed Mins - Afternoon**					

Lo-Act/Med-Sed (ref Hi-Act/Low-Sed)	10.89	4.10	5.53 to 16.27	< 0.001	**0.046**

Hi-Act/Hi-Sed	10.47	2.41	1.67 to 19.28	0.021	

**Sed Mins - Evening**					

Lo-Act/Med-Sed (ref Hi-Act/Low-Sed)	9.81	3.68	4.41 to 15.21	0.001	**0.022**

Hi-Act/Hi-Sed	7.32	1.85	-0.70 to 15.34	0.073	

**MVPA - Core hours**					

Lo-Act/Med-Sed (ref Hi-Act/Low-Sed)	-0.33	-0.46	-1.80 to 1.13	0.648	**0.160**

Hi-Act/Hi-Sed	3.28	3.42	1.34 to 5.22	0.001	

**MVPA - Afternoon**					

Lo-Act/Med-Sed (ref Hi-Act/Low-Sed)	-0.35	-0.62	-1.48 to 0.78	0.536	**0.064**

Hi-Act/Hi-Sed	1.01	1.04	-0.96 to 2.98	0.305	

**MVPA - Evening**					

Lo-Act/Med-Sed (ref Hi-Act/Low-Sed)	-0.52	-1.27	-1.34 to 0.30	0.212	**0.026**

Hi-Act/Hi-Sed	0.41	0.77	-0.66 to 1.47	0.445	

**Weekend**					

**CPM - Core hours**					

Lo-Act/Med-Sed (ref Hi-Act/Low-Sed)	-89.96	-2.46	-163.96 to -15.96	0.018	**0.044**

Hi-Act/Hi-Sed	-83.72	-1.77	-179.28 to 11.84	0.084	

**CPM - Evening**					

Lo-Act/Med-Sed (ref Hi-Act/Low-Sed)	-84.90	-2.19	-163.19 to -6.61	0.034	**0.015**

Hi-Act/Hi-Sed	-112.09	-2.66	-197.16 to 27.01	0.011	

**Sed Mins - Core hours**					

Lo-Act/Med-Sed (ref Hi-Act/Low-Sed)	16.12	2.52	3.18 to 29.06	0.016	**0.036**

Hi-Act/Hi-Sed	5.19	7.50	-9.98 to 20.38	0.493	

**Sed Mins - Afternoon**					

Lo-Act/Med-Sed (ref Hi-Act/Low-Sed)	10.89	4.10	5.52 to 16.27	<0.001	**0.046**

Hi-Act/Hi-Sed	10.47	2.41	1.67 to 19.28	0.021	

**MVPA - Core hours**					

Lo-Act/Med-Sed (ref Hi-Act/Low-Sed)	-2.84	-1.83	-5.98 to 0.29	0.074	**0.055**

Hi-Act/Hi-Sed	-3.05	-1.71	-6.66 to 0.55	0.095	

All models are adjusted for gender, BMI percentile, and Index of Multiple Deprivation for household

The results of the regression models for the weekend periods are also presented in Table 4. The Lo-Act/Med-Sed group obtained an average of 90 fewer counts per minute than the Hi-Act/Low-Sed group during weekend core hours (t = -2.46, p = 0.018). Both the Lo-Act/Med-Sed (-84.9, t = -2.19, p = 0.034) and the Hi-Act/Hi-Sed (-112.1, t = -2.66, p = 0.011) groups had lower mean counts per minute than the Hi-Act/Low-Sed group during weekend evenings with the model only accounting for 1.5% of the variance. The Lo-Act/Med-Sed group recorded more sedentary minutes (16.12, t = 2.52, p = 0.016) than the ref Hi-Act/Low-Sed group during weekend core hours. Both the Lo-Act/Med-Sed (10.89, t = 4.10, p < 0.001) and Hi-Act/Hi-Sed (10.47, t = 2.41, p = 0.021) groups recorded more sedentary minutes than the Hi-Act/Low-Sed group in the afternoon period.

## Discussion

In this paper we have identified three distinct clusters of children related to physical activity and sedentary behaviours. The physical activity patterns of these groups differed in the overall amounts of MVPA, the volume of physical activity, and the time of day when the physical activity occurred. The highest mean minutes of MVPA were obtained by the Hi-Act/Hi-Sed group indicating that the group which spent the greatest amount of time watching TV and playing on computer and video games were also the most active. This finding is comparable to previous research which reported that some youth who spend a lot of time engaged in team sports also spend a lot of time being sedentary [9]. Thus, in terms of identifying who to target for interventions it is important to consider both the physical activity and sedentary behaviours of the participants.

In terms of targeting interventions to specific groups of children, our findings suggest a need to focus on ways to increase the overall physical activity levels of the Lo-Act/Med-Sed group, i.e. children who spend considerable amounts of time watching TV or playing computer games and who are not physically active. Further understanding of this group might be achieved through assessment of the psychosocial characteristics of this group or levels of parental and peer support for physical activity [8,28-30]. Variables such as attitudes to physical activity, perceived physical and social competence, for example, may differ from other groups and provide targets for focusing intervention strategies.

For example, if the Lo-Act/Med-Sed group reported low levels of physical activity enjoyment, an intervention could be specifically tailored to increase the enjoyment of physical activity within this group. Equally, if the group reported low preferences for active behaviours, strategies to increase the preference for these activities could be developed. Thus, in terms of developing more sophisticated and potentially more successful interventions the first step would be to identify the key group characteristics of children and critical time points across the week within which to focus interventions. The second step would to identify the key correlates or predictors of physical activity behaviour during the key time periods for each group and then develop and test targeted interventions in those contextual settings.

During "core hours", the Hi-Act/Hi-Sed group obtain more minutes of MVPA and a higher mean CPM than the active group indicating that they are more active at school. Interestingly, the Hi-Act/Hi-Sed groups spent more time being sedentary in the afternoon period after-school than the Hi-Act/Low-Sed group. When these findings are combined they indicate that the Hi-Act/Hi-Sed group are youth who are active at school but are less active in the period immediately after-school. Thus, a targeted intervention that focuses on increasing the physical activity in the period immediately after-school is likely to have particular resonance for this group.

During core weekend hours the Lo-Act/Med-Sed group engaged in three fewer minutes of MVPA and spent sixteen more minutes being sedentary than the Hi-Act/Low-Sed group. This finding would suggest that developing ways to reduce screen-viewing and engage the Lo-Act/Med-Sed group in physical activity during the weekend is important. Similarly, our data indicate that the Hi-Act/Hi-Sed group have lower CPM during the weekend evenings and higher sedentary counts than the Hi-Act/Low-Sed group, and thus focussing on the physical activity levels of Hi-Act/Hi-Sed group on weekend evenings is also a likely intervention target.

Our analyses have identified three key groups or clusters of children that have different physical activity patterns, together with the time period across the week in which each group is particularly active or sedentary. This provides important insights on how to target and tailor interventions. At this point it is important to recognize that none of the three identified groups of children obtained the government-recommended levels of 60 minutes of MVPA per day. The findings therefore indicate that strategies are needed to increase the physical activity levels of all groups. However, the fact that the Lo-Act/Med-Sed group had the lowest minutes of MVPA per day, as well as lowest mean CPM, suggest such strategies would particularly benefit this group. Additionally, whilst there were no differences in the BMI or deprivation levels of group members, males were more likely to be in the Hi-Act/Low-Sed and Hi-Act/Hi-Sed groups than females, and females were more likely to be in the Lo-Act/Med-Sed group. These findings suggest that the eight questions were able to successfully differentiate between physical activity-related behavioural clusters in these children and could be used as a brief screening questionnaire to identify key groups that could then be appropriately targeted in future interventions.

### Strengths and limitations

This study has provided important evidence that, for children specific clusters of physical activity and sedentary behaviours exist and that these patterns of activity and sedentary behaviours differ across the day for the three cluster groups. The findings thereby provide new information that will facilitate the development of interventions that can be tailored to the needs of each group. The study does, however, have a number of limitations that need to be recognised. It is important to note that participants were included in the overall analyses if they provided a minimum of three days of accelerometer data, in the weekday specific analyses if they provided at least two days of data and in the weekend data if they provided a minimum of one day of data. It has been suggested that more than 3 days of data might be needed to capture the habitual physical activity patterns of children [31,32]. However, as we viewed these analyses as an exploratory first step in understanding the clusters of children's physical activity patterns, we opted for the 3-day inclusion criterion that has been widely used for children and adolescents [33-35] and this decision allowed for the largest possible sample size. It is also important to note that the data are from a cross-sectional survey conducted in a single UK city and therefore replication in more samples is needed to ascertain if the same groupings are obtained. The group classification was also conducted based on four physical activities that occur outside of school and as such these questions are unlikely to be able to capture the key differences in behaviour that occur during the school day.

## Conclusions

The data presented in this paper have shown that for physical activity and sedentary time there are three different clusters of children and the physical activity and sedentary behaviour patterns of these clusters differ across the week. Group membership can be identified from eight brief questions and therefore the questions could be used as a screening mechanism to identify those participants who could potentially benefit the most from increased physical activity. Once membership has been determined, targeted strategies that focus on group-specific periods of low activity could then be implemented and evaluated.

## Competing interests

The authors declare that they have no competing interests.

## Authors' contributions

This study was conceived by RJ, KRF, ASP and JLT. The first draft of the paper and all analyses were produced by RJ. All authors edited the paper and made critical contributions to the paper.
